# See, Touch, Feel, and Express: Achieving Safe and Natural Outcomes With HA Fillers—An International Consensus

**DOI:** 10.1111/jocd.70784

**Published:** 2026-03-07

**Authors:** Atchima Suwanchinda, Hosung Choi, Niamh Corduff, Haiyan Cui, Lindsay Gail Torralba Garcia, Martina Kerscher, Ting Song Lim, Olivia Ong, Je‐Young Park, Danai Praditsuwan, Tuck Wah Siew, Fang‐Wen Tseng, Raymond Wu, Tatjana Pavicic

**Affiliations:** ^1^ Department of Dermatology, Chulabhorn International College of Medicine Thammasat University Bangkok Thailand; ^2^ Division of Dermatology, Department of Medicine, Faculty of Medicine Ramathibodi Hospital, Mahidol University Bangkok Thailand; ^3^ PIENA Aesthetic Medical Clinic Seoul Korea; ^4^ River End Aesthetics Geelong Australia; ^5^ Department of Plastic and Cosmetic Surgery, School of Medicine Tongji Hospital, Tongji University Shanghai China; ^6^ Institute of Aesthetic Plastic Surgery and Medicine Tongji University Shanghai China; ^7^ Wilson & Ayache Medical Aesthetics Clinic Makati City Philippines; ^8^ Division of Cosmetic Science and Aesthetic Dermatology University of Hamburg Hamburg Germany; ^9^ Clique Clinic Kuala Lumpur Malaysia; ^10^ Jakarta Aesthetic Clinic Jakarta Indonesia; ^11^ Apkoo‐Jung Oracle Dermatology Clinic Seoul Korea; ^12^ DIAA Clinic Chiang Mai Thailand; ^13^ Radium Medical Aesthetics Singapore Singapore; ^14^ Everbeaute Medical Aesthetics Taipei Taiwan; ^15^ Asia‐Pacific Aesthetic Academy Hong Kong Hong Kong; ^16^ Private Practice for Dermatology & Aesthetics Dr. Tatjana Pavicic Munich Germany

**Keywords:** cohesive polydensified matrix, consensus, facial rejuvenation, filler, hyaluronic acid, natural outcomes, safety

## Abstract

**Background:**

The concept of “natural outcomes” in filler treatments has been explored in clinical studies and literature, yet remains loosely defined, subjective, and lacks standardized assessment criteria.

**Aims:**

To propose a multidimensional Natural Outcomes Framework to systematically define, assess, and communicate natural outcomes following hyaluronic acid (HA) filler treatment, facilitating attainment of desired results.

**Methods:**

An international aesthetic multidisciplinary panel developed consensus statements and a practical framework for achieving safe and natural outcomes with HA fillers based on insights from a literature review, survey, and expert meeting.

**Results:**

This expert consensus emphasizes safety as a foundational aspect of natural outcomes. The framework extends beyond visual outcomes (“See”) to encompass tactile (“Touch”), experiential (“Feel”) and expressive (“Express”) dimensions of naturalness, which can be assessed by various methods. The panel identified three categories of factors affecting natural outcomes: product, patient, and injector factors. Treatment can be optimized by selecting HA fillers with biomimetic design, suitable rheological properties, and low inflammatory potential; ensuring knowledge and technical competency; individualizing treatment plans; and fostering effective communication. A practical clinical workflow was devised to guide the attainment and assessment of safe and natural outcomes from pre‐treatment to follow‐up.

**Conclusions:**

The Natural Outcomes Framework offers a structured approach to achieving safe and desired outcomes with HA fillers, aligned with this principle: “treat the patient, not the photograph.” It promotes patient‐practitioner alignment on treatment goals and use of appropriate products based on biomimetic design principles, contributing to the attainment of predictable and satisfying results with aesthetic HA filler treatments.

**Abbreviations**
BDD: body dysmorphic disorder
BDDQ‐DV: body dysmorphic disorder questionnaire—dermatology version
CPM: cohesive polydensified matrix
DCQ: dysmorphic concern questionnaire
DIRs: delayed inflammatory responses
EPCs: emergent perceptual categories
FOS: facial overfilled syndrome
GAIS: global aesthetic improvement scale
HA: hyaluronic acid
HCPs: healthcare professionals
OBT: optimal balanced technology
TSST: target‐specific sandwich technique
VAS: visual analog scale

## 1 Introduction

In recent years, there has been a growing demand for aesthetic procedures that deliver natural‐looking outcomes, aligning the current trends in both with patients' desires and healthcare providers' priorities. A 2021 survey by the American Academy of Facial Plastic and Reconstructive Surgery identified “fear of unnatural‐looking results” as one of the top patient concerns [1]. Meanwhile, a survey of 200 Asia Pacific practitioners highlighted natural outcomes and safety as critical priorities in hyaluronic acid (HA) filler treatments [2].

Although several clinical studies and literature have explored the concept of “natural outcomes” in filler treatments, it remains loosely defined, highly subjective, and lacks standardized assessment parameters. Existing definitions in the literature often focus on aesthetic balance—improved skin quality, facial shape, and structure—while achieving harmony between the patient's physical appearance and emotional well‐being [3]. One proposed model defines “natural” as the optimal alignment of beauty, genuineness, and self‐esteem [4]. A study suggested that both static and dynamic facial expressions should be considered in assessing natural‐looking outcomes [5]. From the patient's perspective, natural outcomes involve preserving self‐identity, avoiding unrecognizable changes, and achieving discreet enhancements [6]. Conversely, outcomes such as facial overfill, surface irregularities, disproportionate or distorted appearance have also been described as unnatural‐looking [2]. Notably, most studies seeking to measure natural outcomes have typically relied on visual and photographic assessment, overlooking other critical aspects such as findings on palpation, dynamic facial expressions, and the patient's internal experience and emotions [3, 5, 6, 7].

The subjective nature of “natural outcomes” in aesthetics and the lack of standardized criteria complicate its assessment and hinder the alignment of expectations between patients and aesthetic practitioners. To address this, we propose a “Natural Outcomes Framework”, which aims to systematically define, assess, and communicate natural outcomes following HA filler injections. As with “skin quality”, a subjective and holistic aesthetic concept shown to be evaluable using four emergent perceptual categories (EPCs: skin glow, firmness, surface evenness, and evenness of skin tone [8]), it may likewise be valuable to devise a holistic framework to assess natural outcomes.

This article encapsulates insights gathered from a panel of 14 aesthetic experts who reached consensus on defining and assessing natural outcomes following HA filler treatments. We propose a Natural Outcomes Framework that will serve as a tool to enhance consistency in evaluation, improve communication between healthcare professionals (HCPs) and patients, and establish natural outcomes as an achievable and measurable aesthetic goal. Additionally, in alignment with the framework, we discussed the roles of product selection, patient‐specific factors, and the responsibilities of HCPs in optimizing aesthetic outcomes. A workflow is also proposed to guide the attainment and assessment of safe and natural outcomes throughout the patient journey in HA filler treatments.

## 2 Methods

### 2.1 Conceptualization and Validation of the Natural Outcomes Framework

An extensive literature review, incorporating insights from practitioners' experiences, was conducted to identify items for consideration into the Natural Outcomes Framework. The definitions and contextual understanding of the framework components were further refined through consensus with an international, multidisciplinary panel of 14 aesthetic experts. The experts had a range of professional aesthetic experience and comprised aesthetic physicians, dermatologists, and plastic surgeons from 11 countries/regions (Australia, China, Germany, Hong Kong, Indonesia, Malaysia, the Philippines, Singapore, South Korea, Taiwan, and Thailand). Collectively, the panel possessed an average of 18.1 years of experience in HA filler injections.

A survey questionnaire was designed based on relevant literature and used to gather insights on the following: defining natural outcomes with HA fillers, identifying attributes associated with natural and unnatural results, and understanding the role of safety in the framework. It also solicited experts' opinions on strategies to achieve natural outcomes. The questionnaire was structured into three sections comprising 17 questions, combining closed‐ended formats (such as multiple choice and ranking) with open‐ended items to elicit in‐depth feedback from the experts (Appendix S1). The questionnaire was distributed electronically via email to the panel of 14 aesthetic experts, who were invited to complete it within a specified timeframe. Responses were collected and analyzed to refine the Natural Outcomes Framework based on the panel's insights.

### 2.2 Consensus Statement Recommendations for Natural Outcomes Following HA Filler Treatment

The insights gathered from the questionnaire were used to draft consensus statements. An in‐person advisory board meeting with the 14 aesthetic experts was convened in November 2024 to discuss key topics, refine the consensus statements, and vote on their agreement with each final consensus statement.

Consensus was defined as follows: strong consensus indicated by > 95% agreement, consensus by 75%–95% agreement, majority consent by 50%–75% agreement, and no consensus by < 50% agreement. Only statements and recommendations that achieved consensus or strong consensus were included in the final recommendations.

## 3 Results and Discussion

### 3.1 Conceptualizing Key Elements of “Natural Outcomes”

**FIGURE 1:**
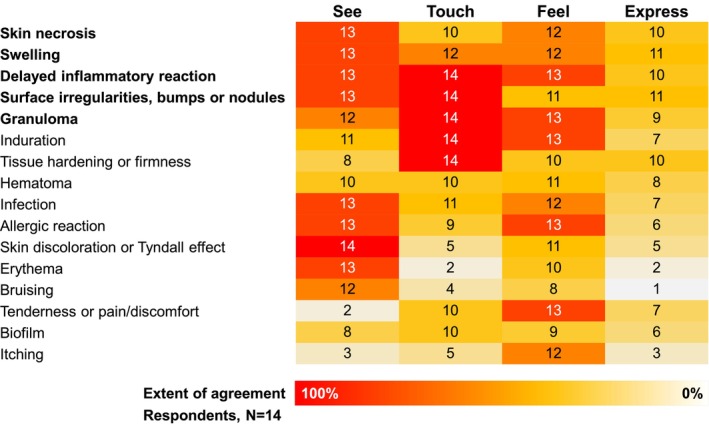
Complications of HA filler treatment can affect multiple elements of natural outcomes. The question posed to the expert panel was: “How does each complication affect these four elements of natural outcomes?” The numbers represent the count of experts (out of 14) who selected each corresponding option. Swelling, granulomas, surface irregularities, bumps and nodules, delayed inflammatory responses, and skin necrosis can adversely affect natural outcomes across all four elements. HA, hyaluronic acid.

The results from the pre‐meeting questionnaire indicated unanimous agreement (14/14, 100%) among the expert panel, who fully endorsed the importance of natural outcomes following HA filler treatments. The panel generally concurred that natural outcomes could be categorized into four key elements: “See, Touch, Feel, and Express”. These elements were selected as they collectively support a holistic approach for assessing naturalness in aesthetic treatments. Unlike conventional assessments that primarily rely on visual observation, this framework integrates tactile, sensory, and dynamic aspects, ensuring a comprehensive evaluation of treatment outcomes. Each element is associated with specific attributes that characterize natural and unnatural outcomes (Table 1). Safety was emphasized as a critical, non‐negotiable, core prerequisite that encompasses all elements of the Natural Outcomes Framework, since complications arising from HA filler treatments potentially affect natural outcomes across all four elements (Figure 1).

**TABLE 1:** Natural/unnatural attributes and assessment within the Natural Outcomes Framework.

	See	Touch	Feel	Express
Natural attributes	Maintains or improves facial symmetry Balanced facial proportions Harmonious facial features Subtle enhancements Realistic aesthetic results Preserves the individual's unique, defining facial features Looks younger/youthful without exaggerated effects Appears refreshed, well‐rested	“Naturally smooth” texture of skin or lips (treated area) upon touch Softness or firmness is consistent with untreated areas “Normal” findings upon palpation, similar to untreated areas Smooth transition between treated and untreated areas	Treated areas feel “normal”, “the same as before” treatment Improved self‐perceived attractiveness Preserved sense of self‐identity and individuality Improved self‐esteem or self‐confidence Positive impact on psychological wellbeing Tolerable injection procedure (e.g., minimal pain, tenderness, itching, bruising, and swelling) Enhanced overall quality of life Comfort in social interactions Satisfaction with the treatment results aligning with personal expectations	Balanced/symmetrical facial expressions Age‐related changes in facial expressions (e.g., wrinkles, jowls) are corrected while maintaining a natural appearance Facial appearance at rest appears relaxed and pleasant Harmonious facial appearance at rest and in motion, expressions are perceived positively (e.g., sparkling, cheerful) Both dynamic and static expressions consistently appear natural and congruent with age Smooth facial movements without stiffness (e.g., seamless smile or frown) Proportional and balanced facial expressiveness Individuality of facial expressions and emotional range are preserved, appearing authentic
Unnatural attributes	Signs of facial overfilled syndrome (FOS): distorted and heavy appearance, “flower horn” foreheads, “sunset” eyes, “chipmunk” cheeks, “witch” chins, and “pillow” faces Inflated cheeks due to unnatural volumization of the anterior cheek Over‐projection of certain facial areas, creating facial asymmetry Procedures result in an artificially uniform look, making individuals appear “generic” Distortion of natural facial contours Over‐exaggeration of youthful features, for example, excessive wrinkle filling or smoothness Mismatch between the treated area and the rest of the face or body, resulting in a noticeable contrast	Palpable signs of swelling or lumps (e.g., masses, nodules, regions of induration, sterile abscesses, granulomas/bogginess/fluctuance) Noticeable surface irregularities or unevenness at or near the treated areas upon touch Unusual firmness at or near the treated areas upon touch Abnormal or altered skin texture or elasticity	Persistent and disturbing physical sensations (e.g., foreign body sensations in the face or lips) Discomfort or pain Heaviness or tightness in the face Not recognizing oneself Appearance feels inconsistent with personal identity or personality Changes perceived as exaggerated or incongruent (e.g., “too young”) Discrepancy between personal style and treatment outcome	Unintended aging‐related negative expressions (e.g., appearing sad, aged, tired) Mismatch between facial expressions and the individual's emotional state Facial asymmetry or disproportionate expressions (e.g., uneven smile) Muscle overactivity or visible muscular tension at rest or during expression Artificial appearance when smiling or speaking Lumps, depressions, or contour irregularities upon facial animation

Abbreviation: FOS, facial overfilled syndrome.

The panel generally agreed that the concept of a “natural outcome” is multifaceted and complex, extending beyond achieving an aesthetically pleasing appearance after a procedure. Rather than merely creating a list of definitions for natural outcomes, which can never be exhaustive, a more effective approach would be to adopt a comprehensive “Natural Outcomes Framework”, incorporating the 4 Key Elements of visual observation (See), palpation (Touch), soliciting patient feedback (Feel), and assessing dynamic facial expressions (Express) to effectively evaluate natural outcomes. This framework adopts a holistic approach, considering not only external perceptions of visible changes but also the patient's physical sensations and internal emotional and psychological state. Furthermore, it recognizes that natural outcomes inherently extend beyond static aesthetic improvements and encompass dynamic and functional characteristics. The goal is to “treat the patient, not the photograph”. To achieve aesthetically pleasing results, it is crucial to customize treatments to the patient's background, preserving each patient's unique facial features and individuality, including ethnicity and cultural aspects of their self‐identity. This ensures an improved, refreshed look without exaggeration, while maintaining the ability to express emotions through facial movements for normal social and interpersonal interactions.

The panel unanimously agreed that safety should always be the top priority when treating patients with HA fillers, regardless of the treatment outcome, reinforcing the principle of “be safe before anything else”. They emphasized that safety and natural outcomes are interrelated, as many side effects from HA fillers lead to some form of unnatural appearance [2]. In fact, the panels' responses underscore that complications such as swelling, granulomas, surface irregularities, bumps and nodules, delayed inflammatory responses (DIRs), and skin necrosis due to a vascular occlusion can adversely affect natural outcomes across all four elements (See, Touch, Feel, and Express) (Figure 1).

### 3.2 Natural Outcomes Framework

**FIGURE 2:**
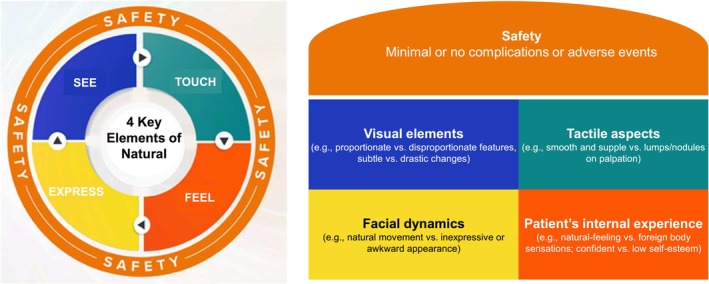
Natural outcomes framework. Safety underpins four key elements: Visual (“See”), tactile (“Touch”), experiential (“Feel”), and expressive (“Express”). The framework consists of four key elements—See, Touch, Feel, and Express—with safety encompassing all elements of natural outcomes. Definitions of each element: SEE: Natural outcomes are achieved when enhancements are subtle and realistic, preserving an individual's unique facial and ethnic features. TOUCH: Natural outcomes are characterized by the absence of palpable swelling, lumps, induration, surface irregularities, or unevenness, as assessed through physical palpation by the clinician and/or patient after the initial filler integration period. FEEL: Natural outcomes reflect to the patient's self‐perception and awareness, encompassing both: (i) the physical absence of a foreign body sensation after a reasonable timeframe; as well as (ii) the psychological and emotional aspect of maintaining self‐identity and individuality while positively impacting self‐esteem. EXPRESS: Natural outcomes preserve the authenticity of the individual's facial appearance and dynamics, without awkward movement and unnatural surface irregularities during animation. SAFETY: A safe product, supported by scientific evidence and a well‐established safety profile, forms the foundation for achieving natural outcomes through safe procedures. HCPs must possess in‐depth facial anatomical knowledge and apply precise injection techniques to ensure the safety of the procedure.

The Natural Outcomes Framework was refined through expert deliberations, and the final version is presented here. This framework provides a structured approach to evaluating naturalness across multiple timepoints (from the immediate post‐treatment phase to long‐term outcomes) according to the 4 Key Elements defining natural outcomes—See, Touch, Feel, and Express—with Safety serving as a foundational prerequisite (Figure 2).

*SEE*: Natural outcomes are achieved when enhancements are subtle and realistic, preserving an individual's unique facial and ethnic features.

*TOUCH*: Natural outcomes are characterized by the absence of palpable swelling, lumps, induration, surface irregularities, or unevenness, as assessed through physical palpation by the clinician and/or patient after the initial filler integration period.

*FEEL*: Natural outcomes should reflect the patient's self‐perception and awareness, encompassing both: (i) the physical absence of a foreign body sensation after a reasonable timeframe; as well as (ii) the psychological and emotional aspect of maintaining self‐identity and individuality while positively impacting self‐esteem.

*EXPRESS*: Natural outcomes preserve the authenticity of the individual's facial appearance and dynamics, without awkward movement and unnatural surface irregularities during animation.

*SAFETY*: A safe product, supported by scientific evidence and a well‐established safety profile, forms the foundation for achieving natural outcomes through safe procedures. HCPs must possess in‐depth facial anatomical knowledge and utilize good injection techniques to maximize the safety of procedures.

### 3.3 Achieving Safe and Natural Outcomes

**FIGURE 3:**
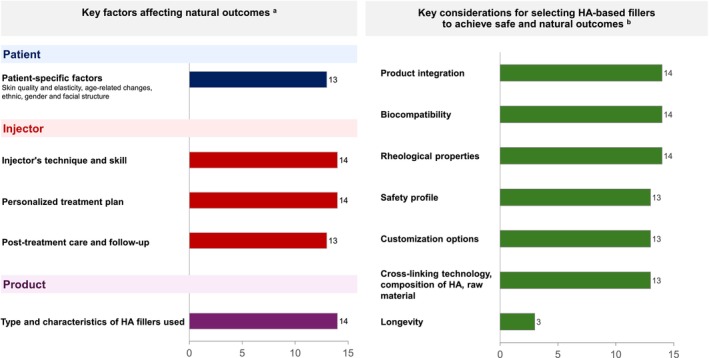
Key factors and considerations for HA filler selection to achieve safe and natural outcomes. ^a^The question posed to the expert panel was: “What are the key factors affecting the achievement of natural outcomes in HA filler treatments?” The complete options included: Patient‐specific factors: Skin quality and elasticity, age‐related changes, ethnic, gender and facial structure; Injector's technique and skill: Knowledge of facial anatomy, precision in injection placement and depth, expertise in adjusting treatment to individual facial features and morphology; Personalized treatment plan: Tailoring the approach to the patients' unique facial characteristics; Post‐treatment care and follow‐up: Proper aftercare to minimize complications, regular assessments to maintain desired outcome; Type and characteristics of HA fillers used: Rheological properties, cross‐linking technology, tissue integration, and risk of DIRs. ^b^The question posed to the expert panel was: “What are your key considerations in selecting HA‐based dermal fillers to achieve a natural outcome?” The complete options included: Product integration: Fillers should integrate seamlessly with the surrounding tissue, avoiding lumps or irregularities and ensuring smooth contours; Biocompatibility: Selecting products that are well‐tolerated by the patients skin with minimal risk of adverse reactions; Rheological properties: Use fillers with appropriate cohesivity, viscosity and elasticity for the specific area being treated to ensure smooth results and natural movement; Safety profile: Prioritizing fillers with a strong track record of safety and minimal side effects (e.g., DIR), to maximize natural, safe, and optimal outcomes; Customization options: Opt for a product range that offers multiple formulations tailored for specific indications or tissue layers, so as to customize a treatment plan according to the patients unique facial anatomy and desired outcomes; Cross‐linking technology, composition and raw materials: Considering the manufacturing process, degree of cross‐linking, and composition of high and low molecular weight HA, as the cross‐linking technology can influence outcomes and the risk of DIR; Longevity: Selecting products that provide long‐lasting results. The numbers represent the number of experts (out of 14) who selected each corresponding option. DIR, delayed immune reaction; HA, hyaluronic acid.

The panel agreed that the factors that significantly affect natural outcomes can be broadly categorized into: (i) product factors (types and characteristics of HA filler); (ii) patient factors (skin quality, anatomy, goals, and expectations); and (iii) injector (practitioner) factors (personalized treatment plan, injector's technique and skill, post‐treatment care and follow‐up) (Figure 3). Addressing each of these factors is essential to maximize safe and natural outcomes in aesthetic treatments.

Focusing on the properties of the filler, key attributes such as tissue integration, biocompatibility, rheological properties, safety profile, customizability, cross‐linking technology, and proportion of low molecular weight HA as starting ingredient all play crucial roles in ensuring safety and achieving natural outcomes (Figure 3).

#### 3.3.1 Product Factors

The selection and correct application of HA fillers are critical for achieving natural results. The panel proposed that a safe filler—and, by extension, a safe treatment—should meet three key criteria, all of which contribute to achieving a natural outcome. First, the filler should integrate seamlessly with the patient's tissues. Second, it should function as a scaffold to support tissue regeneration without triggering significant inflammation or excessive water attraction. Third, its mechanical and rheological properties should ensure even distribution without visible lumps or irregularities, while respecting facial dynamics to ensure that fillers are visually undetectable, imperceptible to touch, and remain in the intended location.

The rheological properties of HA fillers—namely cohesivity, G′ (elastic modulus in shear stress), G″ (viscous modulus in shear stress), Tan delta (a measure of viscoelastic behavior), F_N_ (normal force), E′ (elastic modulus in compression)—and the nature of degradation byproducts are primarily determined by the bioengineering of HA fillers, which encompasses, importantly, the cross‐linking technology, starting HA molecular weight, and post‐crosslinking modifications [9, 10]. These factors, in turn, influence performance characteristics, safety, and, ultimately, treatment outcomes [11].

The panel agreed that to achieve natural outcomes, the HA filler should closely mimic the behavior of native tissue—a principle encapsulated in the concept of “Biomimetic Design”. Fillers adhering to this principle should integrate seamlessly into tissues, providing homogeneous distribution and adaptability to facial movements, and thus achieving natural‐looking outcomes that are visually undetectable and feel smooth to touch, both at rest and during dynamic facial expressions, while ensuring safety with minimal or no DIRs.

The panel reviewed relevant literature and noted that available HA fillers differ in their physicochemical properties, tissue plane adaptability, and inflammatory potential, which may affect the achievement of natural outcomes. Comparative histological and imaging studies have shown that HA fillers with different cross‐linking technologies and structural properties demonstrate distinct behaviors following intradermal and subcutaneous injections. In a study comparing monophasic and biphasic HA fillers implanted intradermally in human volunteers, different cross‐linked formulations exhibited distinct behaviors in the dermis after injection [12]. Similarly, prior evaluations in miniature pigs of 14 HA fillers injected intradermally and subcutaneously demonstrated that monophasic HA fillers, characterized by higher cohesivity and lower viscoelasticity, achieved a more homogeneous distribution than biphasic fillers [13]. Within the broader category of monophasic HA fillers, polydensified formulations, i.e., cohesive polydensified matrix (CPM) gels, have been reported to exhibit homogenous dermal distribution without histological evidence of inflammation or structural tissue alteration; this is consistent with recent integrated physicochemical and histological analyses demonstrating low levels of insoluble particle impurities, as well as good tissue integration without any inflammatory reactions in vivo [12, 14, 15, 16]. In a visual and microscopic study assessing the properties of seven HA fillers with different cross‐linking technologies, variability in cohesivity and microstructural characteristics was observed, with CPM demonstrating the highest cohesivity and an absence of discrete particles on microscopic evaluation [17].

Another key aspect of HA fillers is their safety, which is not only a prerequisite for treatment but also affects the naturalness of the result, as complications can impact all four elements of natural outcomes (See, Touch, Feel, Express) (Figure 2). While HA filler injections are generally considered low‐risk procedures, they can still cause significant vascular and non‐vascular complications, with DIRs being a major concern that is influenced by a HA filler's bioengineering [2]. Although the general recorded rate of DIRs with HA‐based filler treatment is 0.02% [18], the incidence varies among different HA fillers, ranging from 0% to 4.3% [2]. Reports indicate that HA dermal fillers with higher compositions of low molecular weight HA (< 1000 kDa) may be associated with an increased risk of DIRs [2, 19]. A previous Asia Pacific report on practical approaches to achieving safe and natural‐looking results with HA filler treatment recommended that one of the fundamentals for avoiding DIRs is to choose an appropriate HA filler with low immunogenic potential [2].

Several studies have compared the inflammatory responses to HA fillers and demonstrated distinct reactions between products. In one study of two voluminizing HA fillers (based on CPM and Vycross technology) administered subcutaneously either by bolus or retrograde fanning cannula technique in one subject, biopsy findings at 21 days after treatment showed differences in inflammatory response. Notably, in this case, macrophages and giant cells were only observed to be present at the implant site where Vycross HA filler was delivered by retrograde fanning technique [14]. Another study assessed the inflammatory response of HA fillers following intradermal and subcutaneous injections in miniature pigs and found that monophasic fillers elicited lower local inflammatory responses compared to biphasic fillers [13]. Additionally, the fillers with higher viscoelastic properties specifically designed for deeper injections tended to induce slightly higher inflammatory responses than those intended for more superficial layers [13]. These results highlight the importance of selecting high‐quality fillers with low inflammatory potential to achieve predictable outcomes and minimize complications.

To achieve natural outcomes, individualized treatment is essential for HCPs to customize interventions based on patient‐specific needs and aesthetic goals. This requires a range of HA fillers with distinct rheological properties, designed for different facial areas, different anatomical layers, and different indications, alongside corresponding correct injection techniques to achieve optimal results [11]. For example, the Target‐Specific Sandwich Technique (TSST), designed for pan‐facial rejuvenation of Asian patients, utilizes small amounts of different HA fillers with varying rheology and minimal entry points in strategic facial areas. This approach has been shown to yield positive outcomes, enhancing the rejuvenated appearance effectively for Asian patients [20]. Consensus recommendations were previously developed to guide clinicians on the optimal use of the CPM‐HA portfolio, considering its varying rheological properties for specific indications. The recommendations provide guidance on product selection, administration techniques, and injection depths which, when adhered to, allow for optimal treatment outcomes that provide natural aesthetic results while enhancing safety and patient satisfaction [21].

By prioritizing safety, biocompatibility, and innovative design, appropriate use of HA fillers can deliver satisfying outcomes that align with patient‐centered care and aesthetic principles to achieve natural‐looking outcomes [9, 11]. However, even with high‐quality products, it is essential that HCPs have the knowledge and skills to use them correctly. Education plays a crucial role in equipping HCPs with the understanding required to select and apply the appropriate fillers. Training programs should focus on enhancing HCPs' knowledge of product‐specific properties, such as rheology, integration behavior, and safety profiles, ensuring they can make informed decisions to achieve optimal outcomes for their patients [2, 9, 11, 21]. By using a product with a well‐established safety profile, HCPs reduce potential product‐based risks and can focus on proper injection techniques and delivering the HA filler correctly to achieve a good outcome.

#### 3.3.2 Patient Factors

The field of modern aesthetic medicine has transitioned from traditional doctor‐led care to a more consumer‐driven model [22]. In the therapeutic partnership, patients thus play a critical role in achieving natural outcomes, starting with recognizing the importance of setting realistic goals and understanding the value of natural‐looking aesthetic outcomes.

A significant challenge in aesthetic medicine is the commercial and social media influence perpetuating unrealistic beauty ideals, which can lead to unattainable or mismatched expectations [22]. The pursuit of a uniform, flawless appearance—characterized by high cheekbones, full lips, and a contoured jawline—has become a widely promoted beauty standard, often driven by influencers and celebrities [22]. This standard devalues the natural diversity of facial features and erases individuality. Exaggerated aesthetics are often highlighted on digital platforms, likely influenced by the commercial interests of manufacturers aimed at driving higher product sales. Additionally, social media influencers often highlight only ideal outcomes while downplaying risks, complications, and the need for ongoing maintenance. This selective portrayal can lead patients to pursue excessive enhancements, resulting in unmet expectations and dissatisfaction [22].

Shifting the aesthetic industry toward realistic goals focused on achieving subtle, natural outcomes tailored to individual needs and preserving unique identity is crucial. Aesthetic practitioners play a vital role in bridging the gap between patient expectations and realistic results through education. Patients should be encouraged to embrace the “best version of themselves”, prioritizing natural outcome‐focused objectives over trend‐driven ideals. Discussions should emphasize principles such as “less is more, simple is best, and natural is key”. A patient‐centered approach that fosters open communication and shared decision‐making is essential for building trust, managing expectations, and supporting informed choices for optimal treatment.

A separate but equally important concern is identifying patients with body dysmorphic disorder (BDD), a psychiatric condition marked by a preoccupation with perceived physical flaws, causing significant psychological distress and social impairment [23]. BDD is considered a contraindication for aesthetic procedures because the condition can impair patients' decision‐making capacity, and cosmetic enhancements rarely alleviate their concerns and sometimes worsen the level of distress [23]. Left unaddressed, this can result in a cycle of repeated interventions that compromise both aesthetic outcomes and patient well‐being. It is therefore imperative for aesthetic practitioners to identify and manage such conditions before commencing treatment. Practitioners should screen for BDD using brief assessment tools (e.g., Dysmorphic Concern Questionnaire [DCQ] and Body Dysmorphic Disorder Questionnaire‐Dermatology Version [BDDQ‐DV]) and structured interviews, and refer such patients for mental health or multidisciplinary care as appropriate [23].

#### 3.3.3 Injector (Practitioner) Factors

The aesthetic practitioner or injector holds primary responsibility for the choice of product, injection technique, dosage/volume, and treatment area in achieving natural outcomes. It is imperative that HCPs take responsibility for their continued education and stay up to date on advancements in scientific knowledge and clinical best practices.

The root cause of unnatural outcomes caused by injectors often lies in the insufficient understanding of the aging process and its impact on facial anatomical structures, including changes to the skeletal framework and soft tissue distribution. When coupled with improper injection techniques, incorrect product selection, excessive filler volume, and a lack of a personalized treatment strategy, these factors significantly contribute to the occurrence of facial overfilled syndrome (FOS) [24]. FOS is characterized by distinctive features such as a distorted or heavy appearance, making it one of the most prominent form of unnatural outcomes following HA filler injections [24].

To address these issues, the panel emphasized the importance of ongoing training and education for HCPs. HCPs must cultivate a thorough understanding of facial topography, the aging process, and the rheological properties of various products while mastering correct injection techniques for each facial area [24]. The amount of HA filler injected is a critical factor influencing both the aesthetic result and patient safety. Achieving optimal, natural‐looking results and minimizing adverse events relies on selecting the most suitable filler product, understanding how fillers behave in tissue over time, using an appropriate injection volume, employing the correct injection technique, and adopting patient‐customized adjustments [2, 25, 26]. Training programs should prioritize adherence to best practices and promote the highest professional standards of care [24]. Both HCPs and product manufacturers have a shared responsibility to prioritize patient care and well‐being over commercial interests, in alignment with the ethical principle of non‐maleficence [22]. Early recognition of potential issues, such as BDD and FOS, is essential. HCPs should counsel patients against pursuing excessive treatments or procedures when unnecessary. To deliver effective and safe outcomes, HCPs should also recognize that HA fillers cannot address every aspect of the aging process and consider a holistic multimodal approach to aesthetic treatments, integrating various strategies and treatments to achieve optimal results. Furthermore, HCPs should advocate for the maintenance of a healthy extracellular matrix, which plays a vital role in supporting better skin health and enhancing treatment outcomes.

Personalized treatment approaches must form the cornerstone of patient‐centered care [2]. HCPs should avoid focusing solely on achieving “perfect” facial symmetry or adhering to idealized facial proportions or ratios, as these often result in unnatural outcomes. Rather than relying on generic “cookie‐cutter” protocols, HCPs should use their technical expertise and artistic eye to develop individualized treatment plans tailored to the unique needs of each patient and aligned with their expectations.

### 3.4 Consensus

Consensus statements on achieving safe and natural outcomes with HA fillers were developed, discussed, and refined by the panel of experts, with all statements receiving 100% (14/14) unanimous agreement, indicating strong consensus. These statements provide a comprehensive, evidence‐based framework for optimizing HA filler outcomes, emphasizing the use of the Natural Outcomes Framework for assessment and integrating product safety, technical expertise, and ethical patient care to maximize both efficacy and safety (Table 2).

**TABLE 2:** Consensus statements on the achievement of safe and natural outcomes with HA filler.

1	Natural outcomes can be defined by physical and emotional parameters comprising SEE, TOUCH, FEEL, EXPRESS, with SAFETY as an all‐encompassing element/core requisite
2	HCPs should recognize unnatural outcomes (e.g., facial overfilled syndrome [FOS]) as a potential complication of fillers
3	A “safe” filler should deliver natural aesthetic outcomes while minimizing risk of complications or adverse events that lead to unnatural features
4	A “safe” filler should: Utilize a low concentration of pro‐inflammatory, low molecular weight HA as a starting ingredient Be manufactured with a cross‐linking technology that results in a 3‐dimensional structure that resembles native tissue in vivo (biomimetic) Lack of degradation by‐products that increase the risk of immune‐mediated or inflammatory reactions
5	Injection technique is as important as product characteristics for achieving safe and natural outcomes, highlighting the need for good knowledge of facial anatomy, the aging process, and proper practical skills training
6	By using a safe product, HCPs avoid one potential risk and just need to focus on proper injection technique and applying the filler correctly to achieve a good outcome
7	HA fillers designed according to biomimetic principles provide optimal tissue integration and balanced rheological properties, facilitating aesthetic results that fulfill all four elements of natural outcomes and the core requirement of safety
8	HCPs should use the pre‐treatment consultation to set realistic expectations as part of the treatment journey, including assessment for signs/symptoms of body dysmorphic disorder, so as to achieve natural outcomes
9	HCPs should not be fixated on achieving “perfect” facial symmetry or adhering to “ideal” facial proportions/ratios, as these often result in unnatural outcomes
10	Considering the patient's expectations and tailoring a personalized treatment plan during the consultation process are key to achieving natural outcomes
11	HCPs should prioritize natural outcomes over commercial interests when planning aesthetic treatments with HA fillers

*Note:* All experts (14/14, 100%) agreed with the consensus statements, indicating strong agreement. Consensus Strong consensus was indicated by > 95% agreement, consensus by 75%–95% agreement, majority consent by 50%–75% agreement, and no consensus by < 50% agreement.

Abbreviations: FOS, facial overfilled syndrome; HA, hyaluronic acid; HCPs, healthcare professionals.

### 3.5 Practical Application of the Natural Outcomes Framework

The Natural Outcomes Framework is relevant to clinical practice, where it can be readily employed to identify key attributes of both natural and unnatural outcomes. A clear and practical clinical assessment workflow would be valuable to guide the evaluation process, taking into account the timing to assess safety or elements of natural outcomes and assessment methods. The panel agreed that certain complications of injections are easily identified as concerning if they persist beyond 1 week after injection. However, since HA fillers require time to integrate, patients may experience minor discomfort and foreign‐body sensations during the initial period, all of which are expected to resolve over time. Therefore, it is recommended to allow an initial four‐week integration period post‐treatment before assessing the naturalness of aesthetic outcomes.

Regarding assessment methods, for the “See” and “Express” elements, the panel noted that conventional imaging methods (2D and 3D, such as photos and videos) can help document and monitor long‐term treatment outcomes but are limited in capturing subtle signs of unnatural results or complications. HCPs are advised to leverage their clinical expertise, aesthetic judgment, and observational skills during consultations, with imaging technology serving as a complementary tool in assessing natural outcomes. The “Touch” assessment will rely on tactile examination through physician palpation of the patient's face, requiring familiarity with normal facial anatomy and topography. For the “Feel” element, established questionnaires such as Global Aesthetic Improvement Scale (GAIS), FACE‐Q Questionnaire (health‐related quality of life and natural modules) [7, 27], and visual analog scale (VAS) should be used to assess and document patient and practitioner feedback on the treatment outcome. In addition, HCPs should actively inquire about physical symptoms that patients may have experienced, such as tightness, heaviness and foreign‐body sensations.

Another important aspect highlighted by the panel is a 360° evaluation, which considers feedback from patients, HCPs, and family, friends or others. By soliciting feedback from individuals who are familiar with the patient's pre‐treatment appearance and their typical facial expressions and emotional state, the evaluation can be less biased and more reflective of real‐world perceptions. This comprehensive evaluation helps ensure that subtle signs of unnatural results, which may not be immediately noticeable to either the patient or the practitioner, are not overlooked.

**FIGURE 4:**
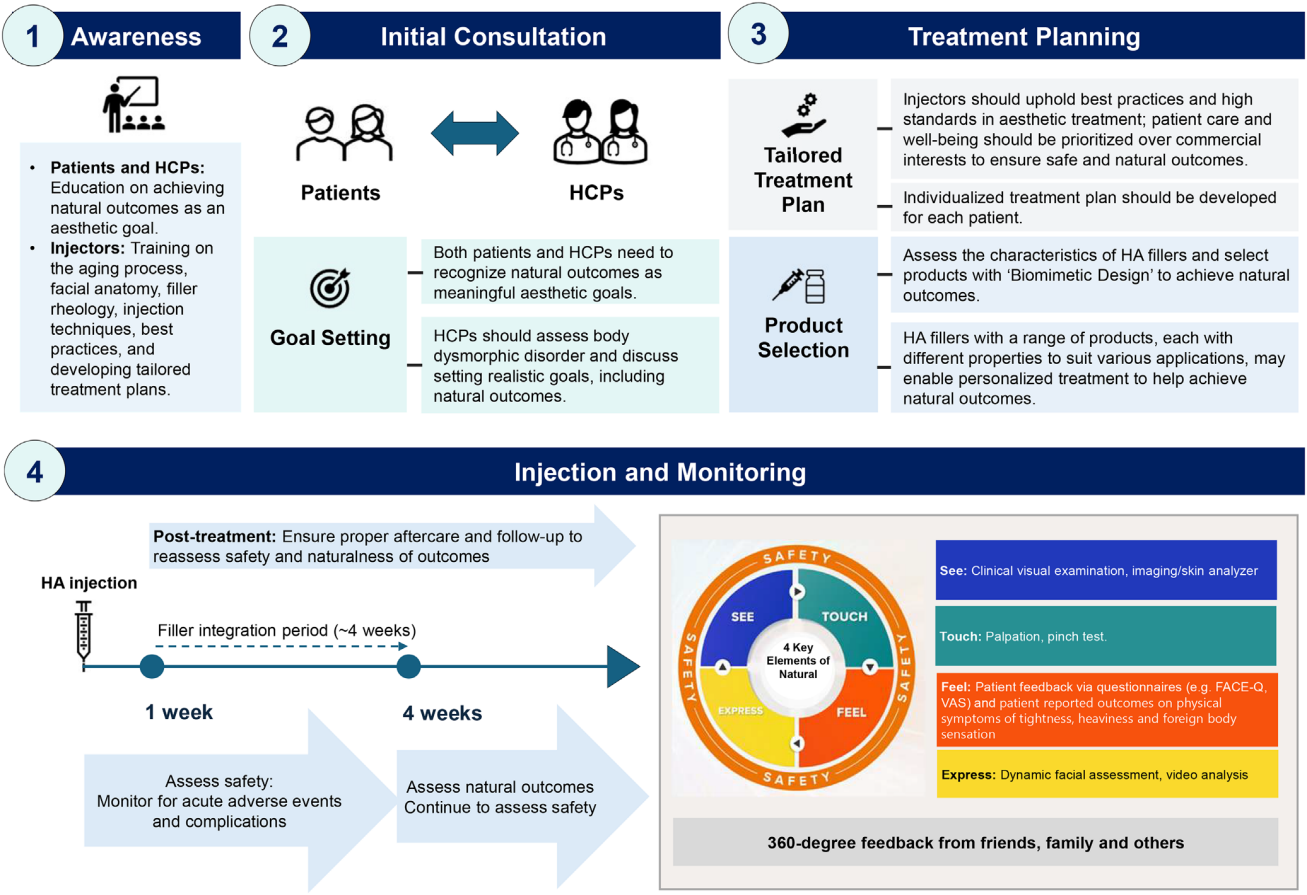
Proposed clinical workflow for achieving safe and natural outcomes. FACE‐Q, Facial Aesthetic Quality of Life Questionnaire; HA, hyaluronic acid; HCP, healthcare professional; VAS, visual analog scale.

A structured workflow for achieving natural outcomes was established based on expert discussions (Figure 4). The process begins with education aimed at increasing awareness of natural outcomes as a fundamental aesthetic goal and enhancing injector competence through advanced training in anatomy, the aging process, filler rheology, injection techniques, and best practices. The initial consultation serves as a foundation for establishing alignment between patients and HCPs, and setting realistic, individualized aesthetic objectives that emphasize natural results. Effective personalized treatment planning requires the development of tailored strategies based on the patient's unique concerns, grounded in ethical principles and a commitment to prioritizing the patient's well‐being. Product selection should be guided by a comprehensive understanding of the characteristics of HA fillers, choosing carefully from a variety of products with distinct properties and bioengineering to attain optimal aesthetic outcomes. Post‐treatment evaluation should include assessments for acute adverse events within 1 week after treatment. A minimum of 4 weeks is recommended to allow for tissue integration before conducting a comprehensive assessment of the naturalness of the outcomes. The key elements of “See, Touch, Feel, Express” under the Natural Outcomes Framework provide a systematic approach to evaluate natural outcomes holistically, incorporating visual, tactile, internal experience (physical and emotional), and expressive parameters. Additionally, a “360° assessment” that includes feedback from individuals familiar with the patient offers further validation of the outcomes. Comprehensive follow‐up care is essential to ensure sustainable results and to address any potential issues that may arise (Figure 4).

## 4 Conclusion

Several factors play a crucial role in achieving safe, natural, and patient‐centered outcomes with HA filler treatments, including product selection, patient characteristics, and practitioner expertise. This paper presents the Natural Outcomes Framework, which incorporates the “See, Touch, Feel, Express” elements of naturalness, with “Safety” as an overarching principle, to guide practitioners in evaluating and achieving the desired outcomes safely, aligning with the principle of “treat the patient, not the photograph”. Achieving optimal and safe outcomes requires alignment between HCPs and patients regarding treatment goals, continued training to enhance injector expertise, and the use of products based on biomimetic design principles. Future application of the framework in clinical practice, together with the incorporation of patient perspectives, will be important for validating and refining its utility. By providing a structured approach, this framework will contribute to more standardized, predictable, and satisfying outcomes in aesthetic HA filler treatments.

## Author Contributions

A.S., H.C., N.C., H.C., L.G.T.G., M.K., T.S.L., O.O., J.‐Y.P., D.P., T.W.S., F.‐W.T., R.W., and T.P.: conceptualization, writing – original draft, writing – review and editing.

## Funding

Merz Aesthetics funded medical writing support.

## Ethics Statement

The authors have nothing to report.

## Conflicts of Interest

A.S.: Speaker honoraria, advisory board participation, travel grants: Merz Aesthetics, Pharma Research. H.C.: Travel grants, speaker honoraria, advisory board participation: Merz Aesthetics, Pharma Research. N.C.: Speaker and clinical advisor to Merz Aesthetics. H.C.: Nothing to declare. L.G.T.G.: KOL & advisory board member for Merz Aesthetics Philippines and Merz Aesthetics APAC. M.K.: Speaker and/or advisor for Merz Aesthetics, Allergan, Neauvia/Matex and Nordberg Medical and is Principal Investigator in clinical trials performed at Hamburg University for Merz Aesthetics, Allergan and Neauvia. T.S.L.: Consultant for Merz Aesthetics. O.O.: No relevant conflicts of interest to disclose. J.Y.P.: Grants from Merz Aesthetics, advisory board member and speaker for Merz Aesthetics. D.P.: Consultant and speaker for Merz Aesthetics. T.W.S.: Consultant for Merz Aesthetics. F.W.T.: Served on advisory boards for and received speaker honorarium from Merz Aesthetics. R.W.: Nothing to declare. T.P.: Consultant and speaker for Merz Aesthetics; performing clinical research for Merz Aesthetics, AAT, AbbVie, and LG.

## Supporting information


**Appendix S1:** Questionnaire for the definition of “natural outcome” after HA‐based dermal filler.

## Data Availability

Data sharing not applicable to this article as no datasets were generated or analyzed during the current study.
