# Supplementary material for: See, Touch, Feel, and Express: Achieving Safe and Natural Outcomes With HA Fillers—An International Consensus
Source: J Cosmet Dermatol. 2026 Mar 7;25(3):e70784. doi: 10.1111/jocd.70784 (PMC12967260; doi:10.1111/jocd.70784)
Supplement: Supplementary file 1 — Appendix S1: Questionnaire for the definition of “natural outcome” after HA‐based dermal filler. [file JOCD-25-e70784-s001.pdf]

Appendix S1

Questionnaire for the definition of ‘natural outcome’ after HA-based dermal filler

Name: ________________
Email: ________________
Clinic: ________________
Country: ________________
Specialty:
☐Aesthetic Physician ☐ General Practitioner ☐ Dermatologist ☐ Plastic Surgeon
Others: ________

Number of years with HA-based dermal filler injection experience: ____________

Background

The American Academy of Facial Plastic and Reconstructive Surgery's 2021 survey reported that one of the top concerns among patients was the fear of an unnatural result following treatment.[1] Despite broad recognition and discussion of ‘natural outcomes’ of filler treatment in the literature, the concept has not been well defined in terms of specific parameters and assessments by practitioners, patients or observers.

A 2021 online survey that included 200 aesthetic practitioners from the Asia Pacific region revealed that safety and natural-looking outcomes were key priorities for HA dermal filler treatments. Unnatural outcomes were described as those with overfilling, surface irregularities, bumps or nodules, facial disproportion, and a distorted appearance. 97% of respondents indicated that achieving a natural outcome for their patients was important. However, an exact definition of a natural outcome was not provided.[2]

Source:
1. American Academy of Facial Plastic and Reconstruction Surgery.
https://www.aafprs.org/Media/Press_Releases/2021%20Survey%20Results.aspx
2. Corduff N et al. Clin Cosmet Investig Dermatol. 2022 Jul 1;15:1213-1223.

Survey Questions

Part 1. Definition of natural outcomes

1. How important is it to create a natural outcome for your patients?

a. Very important
b. Important
c. Neutral
d. Not very important
e. Not at all important

2. Please indicate your level of agreement with the following definitions regarding 'natural outcomes' after aesthetic treatments.

	Strongly Disagree	Disagree	Neutral	Agree	Strongly Agree
Subtle enhancement without noticeable changes					
Aesthetic balance of improved skin appearance (e.g., texture), facial proportions, and emotional well-being (how patients feel about the outcome)					
Aesthetic enhancement while preserving unique facial features and expressions, and maintaining self-identity					
Absence of exaggerated or disproportionate features such as overfilling, surface irregularities, and facial distortion					

Please list or describe in your own words any other attributes/ features that you think contribute to a natural-looking result.

[Free text]

Part 2. 4-element concept of natural outcomes

Perceptions of 'naturalness' are subjective, and standardized criteria for assessing natural outcomes in facial aesthetics are lacking.

To address this gap, a concept based on **four key elements (see, touch, feel, express)** is proposed, with **safety** as a core prerequisite for achieving a natural appearance.

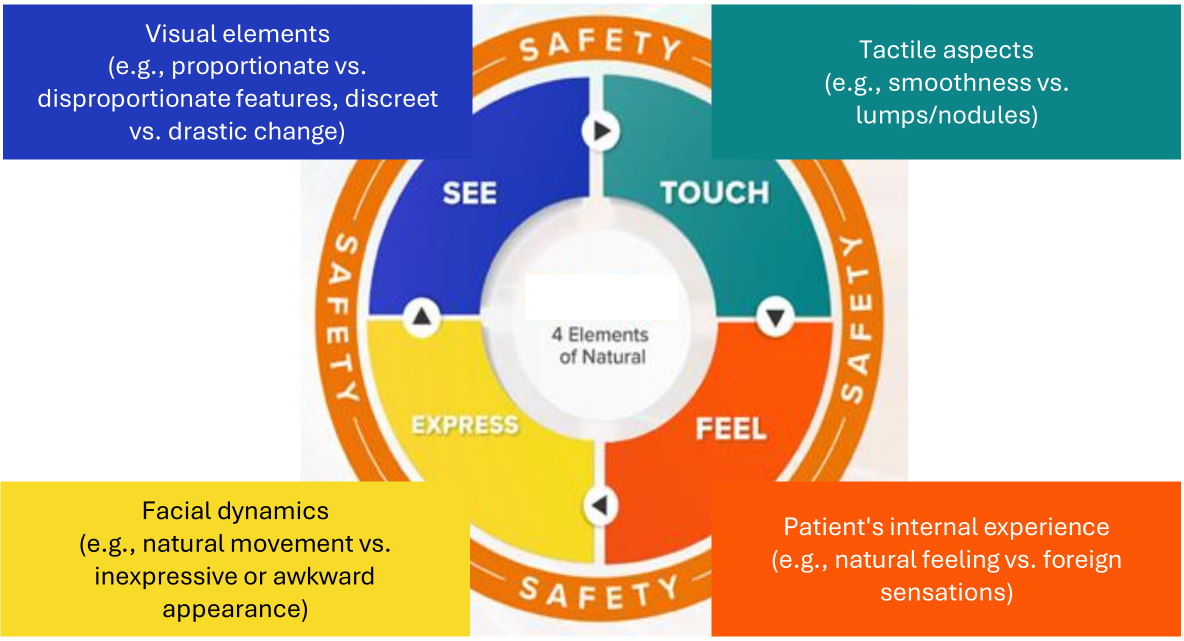

Note: The following questions pertain to the 4-element concept outlined above.

1. Do you agree with the concept of the 4 elements (See, Touch, Feel, Express) in defining a 'natural outcome'?

a. Strongly agree
b. Agree
c. Neutral
d. Disagree
e. Strongly disagree

If you disagree or feel that additional elements should be included, please specify below:

[Free text]

2. Do you agree that using a safe filler is a core and prerequisite element for achieving a 'natural outcome'?

a. Strongly agree
b. Agree
c. Neutral
d. Disagree
e. Strongly disagree

Please let us know why you agree or disagree below:

[Free text]

See

Visual aspects (e.g., proportionate vs. disproportionate features, discreet vs. drastic change)

3. Please select the statements you believe best describe a 'natural' or 'unnatural' outcome related to the 'See' element. [Select all that apply]

Natural

☐ Maintains or improves facial symmetry
☐ Balanced facial proportions
☐ Harmonious facial features
☐ Subtle enhancements
☐ Realistic aesthetic results
☐ Preserves the individual's unique, defining facial features
☐ Looks younger/youthful without exaggerated effects
☐ Appears refreshed, well-rested

Unnatural

☐ Signs of facial overfilled syndrome (FOS): distorted and heavy appearance, 'flower horn' foreheads, 'sunset' eyes, 'chipmunk' cheeks, 'witch' chins, and 'pillow' faces
☐ Inflated cheeks due to unnatural volumization of the anterior cheek
☐ Over-projection of certain facial areas, creating facial asymmetry
☐ Procedures result in an artificially uniform look, making individuals appear 'generic'
☐ Distortion of natural facial contours
☐ Over-exaggeration of youthful features, e.g., excessive wrinkle filling or smoothness
☐ Mismatch between the treated area and the rest of the face or body, resulting in a noticeable contrast

Other important factors:

[Free text]

4. How would you measure 'natural outcome' related to 'See'? [Select all that apply]

☐ Physician/clinician assessed signs of FOS
☐ Patient-reported outcomes (e.g., FACE-Q Aesthetic Natural module items)
☐ Subjective patient and physician/clinician assessment of before and after appearance (e.g., photo/video ± live assessment)
☐ Subjective patient and physician/clinician satisfaction questionnaire (e.g., Global Aesthetic Improvement Scale [GAIS]) or impression on overall natural appearance
☐ Objective measurement of facial symmetry or proportions
☐ Objective assessment of skin appearance (e.g., surface evenness)

Other:

[Free text]

Touch

Tactile aspects (e.g., smoothness vs. lumps/nodules)

5. Please select the statements you believe best describe a 'natural' or 'unnatural' outcome related to the ‘Touch’ element. [Select all that apply]

Natural

☐ ‘Naturally smooth’ texture of skin or lips (treated area) upon touch
☐ Softness or firmness is consistent with untreated areas
☐ ‘Normal’ findings upon palpation, similar to untreated areas
☐ Smooth transition between treated and untreated areas

Unnatural

☐ Palpable signs of swelling or lumps (e.g., masses, nodules, regions of induration, sterile abscesses, granulomas/ bogginess/fluctuance)
☐ Noticeable surface irregularities or unevenness at or near the treated areas upon touch
☐ Unusual firmness at or near the treated areas upon touch
☐ Abnormal or altered skin texture or elasticity

Other important factors:

[Free text]

6. How would you measure ‘natural outcome’ related to ‘Touch’? [Select all that apply]

☐ Patient’s self-rating or perceptions of ‘naturalness’ when touching the treated area
☐ Physician/clinician assessment based on palpation (e.g., pinch test)

Other:

[Free text]

Express

Facial dynamics (e.g., natural movement vs. inexpressive or awkward appearance)

7. Please select the statements you believe best describe a 'natural' or 'unnatural' outcome related to the 'Express' element. [Select all that apply]

Natural

☐ Balanced or symmetrical facial expressions
☐ Age-related changes in facial expressions (e.g., wrinkles, jowls) are corrected while maintaining a natural appearance
☐ Facial appearance at rest appears relaxed and pleasant
☐ Harmonious facial appearance at rest and in motion, expressions are perceived positively (e.g., sparkling, cheerful)
☐ Both dynamic and static expressions consistently appear natural and congruent with age
☐ Smooth facial movements without stiffness (e.g., seamless smile or frown)
☐ Proportional and balanced facial expressiveness
☐ Individuality of facial expressions and emotional range are preserved, appearing authentic

Unnatural

☐ Unintended aging-related negative expressions (e.g., appearing sad, aged, tired)
☐ Mismatch between facial expressions and the individual's emotional state
☐ Facial asymmetry or disproportionate expressions (e.g., uneven smile)
☐ Muscle overactivity or visible muscular tension at rest or during expression
☐ Artificial appearance when smiling or speaking
☐ Lumps, depressions, or contour irregularities upon facial animation

Other important factors:

[Free text]

8. How would you measure 'natural outcome' related to 'Express'? [Select all that apply]

☐ Patient's self-rating or perception of 'naturalness' of their expressions and expressiveness post-treatment
☐ Patient-reported outcomes (e.g., FACE-Q Aesthetic Natural module items)
☐ Subjective patient and physician/clinician assessment of before and after static expressions (e.g., photo ± live assessment)
☐ Subjective patient and physician/clinician assessment of before and after animated/dynamic expressions (e.g., video ± live assessment)

Other:

[Free text]

Feel

Based on patient's internal experience or perception (e.g., natural feeling vs. foreign sensations)

9. Please select the statements you believe best describe a 'natural' or 'unnatural' outcome related to the 'Feel' element. [Select all that apply]

Natural

☐ Treated areas feel 'normal', 'the same as before' treatment
☐ Improved self-perceived attractiveness
☐ Preserved sense of self-identity and individuality
☐ Improved self-esteem or self-confidence
☐ Positive impact on psychological wellbeing
☐ Tolerable injection procedure (e.g., minimal pain, tenderness, itching, bruising, and swelling)
☐ Enhanced overall quality of life
☐ Comfort in social interaction
☐ Satisfaction with the treatment results aligning with personal expectations

Unnatural

☐ Persistent and disturbing physical sensations (e.g., foreign body sensations in the face or lips)
☐ Discomfort or pain
☐ Heaviness or tightness in the face
☐ Not recognizing oneself
☐ Appearance feels inconsistent with personal identity or personality
☐ Changes perceived as exaggerated or incongruent (e.g., 'too young')
☐ Discrepancy between personal style and treatment outcome

Other important factors:

[Free text]

10. How would you measure 'natural outcome' related to 'Feel'? [Select all that apply]

☐ Subjective assessment by patients, e.g., questionnaires evaluating impressions of 'naturalness', satisfaction with appearance, perceived attractiveness, self-esteem, or emotional wellbeing

☐ Time taken for the patient to feel comfortable and resume social interactions without discomfort

☐ FACE-Q questionnaires (psychosocial function, appearance distress, and social function) on the health-related quality of life dimension

☐ Assessment of local tolerability (symptoms such as bruising, redness, pain, tenderness, itching, and swelling)

☐ Evaluation of overall improvement through patient and physician/clinician questionnaires (e.g., Global Aesthetic Improvement Scale [GAIS])

☐ Subjective patient interviews and feedback on the overall impact of the treatment on quality of life

Other:

[Free text]

Safety

1. What are the most concerning complications of HA-based dermal filler? [Rank from 1 to 8]

□ Injection site reactions (e.g., swelling, bruising, redness, pain or tenderness, and itching)
□ Surface irregularities (e.g., nodules/ bumps, induration, tissue hardening/firmness
□ Infection (e.g., skin infection, abscess)
□ Allergic reaction
□ Delayed inflammatory reaction (DIR)/granuloma
□ Biofilm
□ Skin discoloration/Tyndall effect
□ Vascular complications (e.g., skin necrosis, blindness)

2. How does each complication affect these 4 elements of natural outcomes? [Select all that apply]

	See	Touch	Express	Feel	Not applicable
Erythema					
Swelling					
Bruising					
Itching					
Hematoma					
Granuloma					
Tenderness or pain/discomfort					
Surface irregularities, bumps or nodules					
Induration					
Tissue hardening or firmness					
Infection					
Allergic reaction					
Delayed inflammatory reaction					
Biofilm					
Skin discoloration/ Tyndall effect					
Skin necrosis					

3. In your own words, explain how safety influences natural outcomes.

[Free text]

Part 3. How to achieve natural outcomes

1. What are the key factors affecting the achievement of natural outcomes in HA filler treatments? [Select all that apply]

☐ Injector’s technique and skill: knowledge of facial anatomy, precision in injection placement and depth, expertise in adjusting treatment to individual facial features and morphology

☐ Patient-specific factors: skin quality and elasticity, age-related changes, ethnic, gender and facial structure

☐ Type and characteristics of HA filler used: rheological properties, cross-linking technology, tissue integration, and risk of DIRs

☐ Personalized treatment plan: tailoring the approach to the patient’s unique facial characteristics

☐ Post-treatment care and follow-up: proper aftercare to minimize complications, regular assessments to maintain desired outcome

Other considerations:

[Free text]

2. What are your key considerations in selecting HA-based dermal fillers to achieve a natural outcome? [Select all that apply]

☐ Rheological properties: use fillers with appropriate cohesivity, viscosity and elasticity for the specific area being treated to ensure smooth results and natural movement

☐ Longevity: selecting products that provide long-lasting results

☐ Biocompatibility: selecting products that are well-tolerated by the patient’s skin with minimal risk of adverse reactions

☐ Product integration: fillers should integrate seamlessly with the surrounding tissue, avoiding lumps or irregularities and ensuring smooth contours

☐ Cross-linking technology: considering the manufacturing process, degree cross-linking, and composition of high and low molecular weight HA, as the cross-linking technology can influence outcomes and the risk of DIR

☐ Customization options: opt for a product range that offers multiple formulations tailored for specific indications or tissue layers, so as to customize a treatment plan according to the patient’s unique facial anatomy and desired outcomes.

☐ Safety profile: prioritizing fillers with a strong track record of safety and minimal side effects (e.g., DIR), to ensure natural, safe, and optimal outcomes

Other considerations

[Free text]

Thank you for your time

END OF SURVEY

If you have any questions or require additional information/assistance during the survey, please contact the survey team at kelen.alim@tech-observer.com.
